# Social and Environmental Neighborhood Typologies and Lung Function in a Low-Income, Urban Population

**DOI:** 10.3390/ijerph16071133

**Published:** 2019-03-29

**Authors:** Jamie L. Humphrey, Megan Lindstrom, Kelsey E. Barton, Prateek Man Shrestha, Elizabeth J. Carlton, John L. Adgate, Shelly L. Miller, Elisabeth Dowling Root

**Affiliations:** 1 Department of Mechanical Engineering, University of Colorado Boulder, 427 UCB, Boulder, CO 80309 USA; prateek.shrestha@colorado.edu (P.M.S.); shelly.miller@colorado.edu (S.L.M.); 2 Department of Geography and Division of Epidemiology, The Ohio State University, 1036 Derby Hall, 154 North Oval Mall, Columbus, OH 43210, USA; Lindstrom.42@osu.edu (M.L.); Root.145@OSU.edu (E.D.R.); 3 Department of Environmental and Occupational Health, Colorado School of Public Health, University of Colorado, Anschutz Medical Campus, 13001 E 17th Place, Campus Box B119, Aurora, CO 80045, USA; kelsey.barton@ucdenver.edu (K.E.B.); elizabeth.carlton@ucdenver.edu (E.J.C.); john.adgate@ucdenver.edu (J.L.A.)

**Keywords:** social and environmental determinants of health, neighborhoods and health, latent profile analysis

## Abstract

Consensus is growing on the need to investigate the joint impact of neighborhood-level social factors and environmental hazards on respiratory health. This study used latent profile analysis (LPA) to empirically identify distinct neighborhood subtypes according to a clustering of social factors and environmental hazards, and to examine whether those subtypes are associated with lung function. The study included 182 low-income participants who were enrolled in the Colorado Home Energy Efficiency and Respiratory Health (CHEER) study during the years 2015–2017. Distinct neighborhood typologies were identified based on analyses of 632 census tracts in the Denver-Metro and Front Range area of Colorado; neighborhood characteristics used to identify typologies included green space, traffic-related air pollution, violent and property crime, racial/ethnic composition, and socioeconomic status (SES). Generalized estimating equations were used to examine the association between neighborhood typology and lung function. We found four distinct neighborhood typologies and provide evidence that these social and environmental aspects of neighborhoods cluster along lines of advantage/disadvantage. We provide suggestive evidence of a double jeopardy situation where low-income populations living in disadvantaged neighborhoods may have decreased lung function. Using LPA with social and environmental characteristics may help to identify meaningful neighborhood subtypes and inform research on the mechanisms by which neighborhoods influence health.

## 1. Introduction

A large body of work has assessed the respiratory health impacts of social factors and environmental hazards, although this work has generally appeared in separate disciplinary literatures. Consensus is growing on the need to investigate their complex and interdependent effects, as they are often spatially correlated, may operate through common biological mechanisms, and may act jointly to affect health [1,2,3,4,5,6,7]. Recent evidence suggests that the cumulative effects of social factors and environmental hazards work to produce health disparities in urban settings [2,8]. For example, some report that the association between air pollution and asthma is stronger in children who either have high exposure to community violence or live in neighborhoods characterized by socioeconomic deprivation or racial segregation [9,10]. The accumulation of neighborhood social disadvantage and disproportionate exposure to environmental hazards create a series of complex and interdependent mechanisms that may lead to disparities in respiratory health.

Many epidemiological studies account for socioeconomic factors by treating them as confounders of the relationship between health and environmental exposures [3]. But this assumes that health risks from socioeconomic conditions are constant or proportional across different groups or communities and, in effect, independent from one another [11]. However, racial, social, and economic factors also lead to the unequal burden of physical, chemical, and biological exposures within and across communities [3,12]. Numerous studies have documented the disproportionate location of hazardous waste sites, industrial facilities, and other locally undesirable and potentially polluting land uses in socially disadvantaged or racial/ethnic minority neighborhoods [2,13,14]. At the same time, lower-income communities often have an excess of health-damaging factors and a shortage of health-promoting amenities [2,12]. These inequalities differentially shape an individual’s or community’s ability to cope, mitigate, or adapt when exposed to the constellation of hazards present in urban areas [15,16]. Thus, residents of two communities with similar socioeconomic conditions may have different levels of environmental exposure because of their capacity to modify behaviors, improve housing, or receive preventive health services.

To date, most research on the combined respiratory health effects of environmental exposures (e.g., traffic-related air pollution) and social factors (e.g., violence, poverty) has focused on asthma or asthma-like symptoms; little is known about how the joint effects of neighborhood-level social factors and environmental hazards work together to impact lung function. This study takes advantage of the low-income sampling scheme and objective health data from the Colorado Home Energy Efficiency and Respiratory Health (CHEER) study to examine the impact of neighborhood-level social factors and environmental hazards on lung function in a low-income, urban population. We apply latent profile analysis (LPA) to explore how social and environmental characteristics cluster together to create distinct neighborhood typologies that simultaneously capture sociodemographic inequality and environmental risk. Because the CHEER study sampled only low-income homes, we are able to estimate associations between respiratory health and neighborhood typologies within a population that, at the individual level, has a similar socioeconomic status (SES). By restricting the sample to low-income populations, we focus on individuals at highest risk of respiratory health illness. We hypothesize that: (1) The social and environmental aspects of neighborhoods will cluster along lines of advantage/disadvantage, and (2) that individuals living in neighborhoods characterized by poorer physical and socioeconomic environments have worse lung function than their counterparts living in neighborhoods characterized by resource-rich social and physical environments.

## 2. Materials and Methods

### 2.1. Participants

This study utilized data from the CHEER study, which was designed to assess the relationship between housing characteristics related to energy efficiency and respiratory health in low-income, urban households in the Denver-Metro and Front Range area of Colorado. Detailed data on the study design are available elsewhere [17]. Briefly, 303 CHEER participants were recruited from low-income, non-smoking households from the cities of Denver, Aurora, Boulder, Loveland, and Fort Collins over 18 months, from 15 October 2015 to 15 April 2017. Households were recruited through partnerships with agencies that provide housing or work to improve energy efficiency in low-income homes. Income eligibility was different across the partner agencies, but all used thresholds well below the US Department of Housing and Urban Development (HUD) definition of low income: “A household whose income does not exceed 80 percent of the median income of the area, as determined by HUD, with adjustments for smaller or larger families.” [18] Eligible households were those that: (1) met the low-income criteria defined by the participating agency, (2) contained only residents that were reported non-smokers, (3) contained residents that had lived in the home for at least six months, and (4) were a single-family home, duplex, or townhome with no direct air exchange vents in between each unit. Upon enrollment, a three-person study team conducted a two-hour home visit to assess energy efficiency, household characteristics, individual socio-demographics, subjective measures of respiratory health, and lung function tests. All homes were geocoded based on residential address and assigned to the corresponding census tract. All participants provided assent and/or written informed consent. The study protocol was approved by the University of Colorado Boulder Institutional Review Board (protocol #14-0734).

### 2.2. Measures

#### 2.2.1. Respiratory Health

Lung function tests were administered to eligible participants using an EasyOne Plus peak flow portable spirometer [19]. Participant age, sex, standing height, weight, self-reported race/ethnicity, and contraindicators related to spirometry testing were recorded. Participants were not eligible to attempt spirometry if they were younger than eight years of age or had certain medical conditions, including Chronic Obstructive Pulmonary Disorder (COPD), recent major surgery, or other contraindicators detailed in the American Thoracic Society (ATS) guidelines [20]. Participants were coached by field technicians to perform at least three, and up to eight, spirometric maneuvers per test. A pulmonologist blind to exposure status reviewed all maneuvers to determine the acceptability of each curve. Participants were included in our analysis if they achieved three or more acceptable curves per ATS guidelines. Among the 303 CHEER participants, 263 completed pulmonary function tests. Of those, 193 (73%) achieved at least three acceptable forced expiratory volume in 1 second (FEV_1_) and forced vital capacity (FVC) tests. For each participant, the highest FEV_1_ and FVC curves were selected to use in analyses and derive the FEV_1_/FVC ratio per ATS guidelines [20]. Spirometry curves were transformed into standardized z-scores using the 2012 Global Lung Initiative equations (GLI) [21].

Basic demographic information was collected from each participant, and smoking history was assessed via a questionnaire administered to all participants over age 13 using a standard survey instrument [22].

#### 2.2.2. Household Characteristics

We used a multi-point depressurization blower door test to measure the air-tightness of the building envelope, which was inputted to an infiltration model used to estimate the annual average infiltration rate (AAIR) for each CHEER home. The AAIR is an estimate of home ventilation by infiltration and equals the volume of indoor air replaced by outdoor air every hour by infiltration, averaged over the entire year [23]; see Carlton et al., 2019 [17] for a detailed description of this measure. The presence of a gas stove was used as a surrogate for NO_2_ exposure, as studies have found that homes with gas stoves have greater NO_2_ concentrations compared to homes with electric stoves.

#### 2.2.3. Neighborhood Level Measures

##### Green Space

Green space was quantified using the mean normalized difference vegetation index (NDVI). USGS Landsat8 satellite [24] imagery data with a 30 × 30 m resolution was obtained in June 2016. The NDVI measured the surface greenness, or relative biomass of the study region, by using the equation NDVI = (IR − R)/(IR + R), where IR represents the values of the infrared band and R represents the values of the red band. The NDVI was calculated for each pixel, then census tracts were overlaid on the grid in a geographic information system (GIS) to create a weighted-average NDVI for each census tract.

#### 2.2.4. Traffic-Related Air Pollution

Census tract annual estimates of 2015 traffic-related fine particles (PM_2.5_) and nitrogen oxides (NO_x_) were developed using the Community LINE Source Model (C-Line) [25], a reduced-form dispersion modeling program developed by the US Environmental Protection Agency (EPA). The web-based platform uses emission factors, road network data, traffic activity data, and meteorological data to model traffic-related air pollution on a neighborhood scale [26]. The concentration estimates only account for pollution produced by traffic; other sources of pollution are not included. C-Line sources its input data from the EPA, the National Weather Service, and the Federal Highway Administration.

#### 2.2.5. Violent and Property Crime Rates

All point-level arrest data for 2015–2016 were obtained primarily via open records requests and were collected from the counties of Arapahoe, Adams, Boulder, Broomfield, Denver, Jefferson, and Larimer, as well as the cities of Boulder and Longmont. Data were coded as violent, property, or other crimes according the FBI Uniform Crime Reporting program [27]. Violent and property crimes were geocoded, spatially joined to census tracts using a GIS, and summed to obtain the total number of violent and property crime events for each census tract. Crime rates per 1000 population were calculated using the following formula: (total number of crimes/census tract population) × 1000. Census tract population estimates were obtained from the American Community Survey’s 2011–2015 five-year estimates [28].

#### 2.2.6. Socio-Demographic Data

Socio-demographic data were obtained from the 2011–2015 American Community Survey [28]. Measures included the proportion of non-Hispanic white, non-Hispanic black, Hispanic, residents below the federal poverty line, residents with a bachelor’s degree or higher, and homeowners. Median household income was also extracted.

### 2.3. Analytic Strategy

The complex interdependent relationships between neighborhood-level social factors and environmental hazards demand a holistic approach to understanding neighborhood environments. Latent profile analysis (LPA) is a method that allows for a multidimensional and relational approach to neighborhoods and health by exploring how covarying characteristics combine to produce distinct neighborhoods [29,30,31]. LPA empirically identifies homogeneous subgroups based on common characteristics, creating mutually exclusive neighborhood typologies [29]. Traditionally, neighborhood variables have been examined in isolation, which may result in an over-simplification of—and obscure heterogeneity between—neighborhood characteristics [29,32]. LPA is a neighborhood-centered approach that allows the emergence of neighborhood types represented in the data—classifying neighborhoods holistically and configurationally [29,31,32]—and does not require the researcher to pose a priori categories [29,33]. This type of finite mixture modeling empirically determines whether interrelationships exist among observed variables that explain an underlying (latent) construct [29,33].

LPA was conducted using 632 census tracts from the Denver-Metro and Front Range of Colorado. LPA uses continuous indicator variables to create a categorical latent variable of mutually exclusive neighborhood typologies using maximum likelihood estimation [30,33,34]. Neighborhood types are constructed based on differences in means and posterior probabilities. We used the R MCLUST package [35] to test for latent profile solutions of one to five classes. Models were compared to examine the best fit for the data; the number of latent profiles was determined by examining the Bayesian information criterion (BIC), sample size, a bootstrap likelihood ratio test (LRT), and interpretability. The LRT tests for k vs k-1 profiles; a significant LRT test (*p* < 0.05) indicates that the higher-class solution (e.g., four class vs. three class) is a better fit for the data. Each census tract was assigned to the latent profile for which the posterior probability (the probability of membership to in a particular latent profile) was highest [29,30]. Such probabilistic strategies bring the risk of classification error, but the posterior probabilities resulting from our LPA were quite high (e.g., 93% of census tracts were assigned to a latent profile with posterior probabilities at or above 0.90). The data were then exported and all subsequent analyses were conducted in SAS 9.4 [36] (SAS Institute Inc., Cary, NC, USA). We used analysis of variance (ANOVA) to assess mean differences in neighborhood characteristics, outcomes, and covariates across neighborhood typologies; categorical variables were assessed using a chi-square test of difference.

We used generalized estimating equations (GEE) to model FEV_1_, FVC, and FEV_1_/FVC z-scores as a function of living in a specific neighborhood typology; each outcome was modeled separately. Models were adjusted for covariates known to be associated with lung function including sex, age, race/ethnicity, smoking status, and socioeconomic status. Race/ethnicity was operationalized as a binary (white vs non-white) variable. Smoking status was operationalized as a binary metric (former vs never); current smokers were not eligible to participate in CHEER. Socioeconomic status was classified based on the educational attainment of the head of the household. Models were also adjusted for annual average household infiltration rate (an estimate of ventilation rate), the presence of a gas stove (a proxy for indoor exposure to NO_2_), and a binary indicator identifying whether the spirometry test was administered during flu season (defined by the Centers for Disease Control and Prevention [37]). Because individuals are nested within households, we used household-level interchangeable correlation matrices to account for clustering within households and adjust standard errors [38].

## 3. Results

### 3.1. Neighborhood Typologies

The LPA revealed that the 632 census tracts across the study area clustered into four distinct neighborhood typologies. The four-class solution best fit the data, and the ANOVA tests (Table 1) demonstrated that all social and environmental input variables were significantly different across the four neighborhood profiles. Table 1 displays the variables’ means by neighborhood typology. We labeled the first profile *Mixed race, high poverty, moderately poor physical environment*. This neighborhood typology was the most impoverished, and had high concentrations of Black and Hispanic residents, as well as moderately high crime rates and air pollution. The *Mixed race, high poverty, moderately poor physical environment* neighborhood typologies were, generally, the first ring of neighborhoods outside of an urban core. The second profile was labeled *Hispanic and white, high crime, moderate poverty, poor physical environment*. This neighborhood type was characterized by the highest levels of air pollution and crime, the lowest levels of green space, moderate levels of poverty, a highly educated population, and a mix of white and Hispanic residents. The *Hispanic and white, high crime, moderate poverty, poor physical environment* neighborhoods were located close to major roads, highways, interstates, and urban cores. Likewise, they were some of the fastest growing areas of the study region, and are likely picking up the rapid gentrification occurring in the Denver-Metro area [39].

We labeled the third profile *Hispanic and white, moderate poverty, moderate physical environment* (Table 1). This type of neighborhood was characterized by moderately-high levels of poverty, but also homeownership, white and Hispanic residents, low levels of crime and air pollution, and high amounts of green space. These neighborhoods, generally, represent the suburban areas across the Denver-Metro and Northern Front Range of Colorado. The final profile was labeled *White, wealthy, good physical environment*. This profile was characterized largely by white residents with the highest levels of green-space and wealth indicators, and the lowest levels of poverty, air pollution, and crime. These neighborhoods are located close to universities or the wealthy suburbs in the Denver-Metro area. In sensitivity tests (not shown), we found that the neighborhood typologies were robust to the input variables as long as the core domains remained in the model. That is, as long as at least one measure of green space, traffic-related air pollution, crime, income/poverty, wealth, or education were included, the same four typologies were estimated in the LPA.

### 3.2. Neighborhood Typologies and Lung Function

The CHEER analytic sample was comprised of 182 low-income individuals with three or more acceptable spirometric curves. Distributions and test for differences between neighborhood typologies in the outcome and control variables are presented in Table 2. ANOVA tests revealed that FEV_1_, FVC, and FEV_1_/FVC z-scores did not vary significantly by neighborhood typology (Table 2). Household infiltration rate (*F* = 5.38, *p* = 0.002), race/ethnicity (*χ^2^* = 21.26, *p* < 0.001), and socioeconomic status (SES) (*χ^2^* = 17.67, *p* < 0.001) were significantly different across neighborhood typologies.

Table 3 presents GEE models examining the association between living in social and environmental neighborhood typologies and FEV_1_, FVC, and FEV_1_/FVC z-scores. The unadjusted models revealed no significant association between neighborhood typology and lung function. After covariate adjustment, living in a *Mixed race, high poverty, moderate-poor physical environment* neighborhood was associated with a decrease in FEV_1_/FVC (*β* = −0.33, SE = 0.15, *p* = 0.03) relative to living in *White, wealthy, good physical environment* neighborhoods. Living in a *Hispanic and white, moderate poverty, moderate physical environment* was marginally associated with a decrease in FEV1/FVC (*β* = −0.37, SE = 0.20, *p* = 0.07). No significant effects were found for respondents living in *Hispanic and white, high crime, moderate poverty, poor physical environment* neighborhoods or for FEV_1_ or FVC.

## 4. Discussion

This study examined the relationship between social factors and environmental hazards within neighborhoods and their relationship with lung function. Using data from the CHEER study, we went beyond prior research that focused on either social factors or environmental hazards, and incorporated the constellation of characteristics that make up these different neighborhood environments. We found that census-tract-level green space, traffic-related air pollution, violent and property crime, racial/ethnic composition, and socioeconomic status clustered into four distinct neighborhood profiles across the Denver-Metro and Northern Front Range of Colorado. Our findings revealed that the health-enhancing or health-threatening aspects of social and environmental neighborhood contexts accumulated within typologies. For example, neighborhood typologies characterized by low SES levels also had high crime rates, high traffic-related air pollution, and low green space, while neighborhood typologies with high SES had the most green space, low traffic-related air pollution, and low crime rates.

This accumulation of social and environmental (dis)advantages in neighborhoods may be viewed as an inequality generating process because, most often, disadvantage increases exposure to hazards, while advantage increases exposure to opportunity [40,41]. While not discounting the importance of individual-level behavior, genetic factors, and so on, accumulation of neighborhood-level (dis)advantages highlight the social factors and environmental hazards that may shape the social patterning that affects lung function within individuals from neighborhoods across the Denver-Metro and Northern Front Range of Colorado. Neighborhood social factors, including crime rates, racial/ethnic composition, and socioeconomic status may exacerbate vulnerabilities to the health effects of environmental hazards through multiple mechanisms, including limiting opportunities for socioeconomic mobility, access to health-promoting resources, exposure to safe areas, and access to nutritious food and quality health care [2,3,7,12,42]. This is particularly important in this study as the CHEER sample was recruited from low-income households. Previous research has suggested that, relative to higher SES individuals, the health of their low-SES counterparts will be particularly worse off if they reside in disadvantaged neighborhoods because of (1) compounding sources of disadvantage at the individual and neighborhood level, and (2) a reliance on the collective health-enhancing resources available in the neighborhood [43].

We hypothesized that individuals living in neighborhoods characterized by poorer physical and socioeconomic environments would have worse lung function. Results from GEE models provide limited support for this hypothesis and suggest that these empirically identified neighborhoods may be capturing a larger process related to both environmental exposures and socioeconomic inequalities that impacts lung function. When comparing spirometry outcomes across individuals from these different neighborhood profiles, we found suggestive evidence that residing in *Mixed race, high poverty, moderate-poor physical environment* and *Hispanic and white, moderate poverty, moderate physical environment* neighborhoods was associated with reduced lung function as measured by FEV_1_/FVC. Given the relatively small sample size and large confidence intervals (Table 3), the borderline significant associations could be a result of measurement error, or represent a true null effect. We believe our outcome measurement is reliable. Lung function tests were administered by field technicians who followed strict protocols and were trained with the National Institute of Occupational Safety and Health Spirometry Training Program [44]. Moreover, a pulmonologist blind to exposure status reviewed all maneuvers to determine the acceptability of each curve, and participants were included in analyses only if they met ATS acceptability guidelines [20]. Given the direction of the coefficients and borderline p-values, we believe these results are indicative of a true association between FEV_1_/FVC and residential neighborhoods, but the precision of the association is mitigated by the fact that we have a small sample size and wide confidence intervals.

These findings are in line with previous research that examined, to a lesser degree, the confluence of neighborhood social and environmental factors on respiratory health. For example, Kranjac et al. [10] used LPA to classify census tracts in Houston, TX into distinctive communities of racial and sociodemographic characteristics, then explored the associations between neighborhood profiles, census-tract-level air pollution, and childhood asthma. They found that children who lived in disadvantaged or middle-class (relative to advantaged) neighborhoods were exposed to more air pollution, and were more likely to be diagnosed with asthma. Other studies examining the social susceptibility to respiratory health effects of environmental hazards have found, for example, stronger associations between air pollution and asthma in higher socioeconomically deprived [45,46] or higher-crime neighborhoods [9], further supporting the evidence that the cumulative effects of social factors and environmental hazards work to produce health disparities in urban areas.

The complex interdependent relationships between neighborhood-level social and environmental characteristics demand a comprehensive approach to understanding the impact of neighborhood context on health. Yet, methodologically, challenges exist in how to evaluate and characterize the combined health effects of multiple environmental hazards and social factors. For instance, in studies examining the interdependent relationship between green space, air pollution, and health, researchers are continually refining methods to ascertain whether green space is a mediator, modifier, or confounder on the air pollution–health causal pathway [47]. These kinds of complex relationships exist across the spectrum of neighborhood components and may lead to issues in modeling and interpretation of neighborhood health effects studies [12]. Rather than trying to tease apart the relative influence of each neighborhood factor, latent profile analysis provides a unique perspective on the delineation of neighborhoods within communities by holistically exploring the environments to which people are exposed. The LPA for this study revealed four distinct classes; these classes were constructed and shown to not only tie together social and environmental similarities in neighborhoods, but also spatial similarities. LPA sufficiently answers a call that has been made for creative thinking about the nature of neighborhood social [and environmental] contexts, and reaches further to add a dimension of understanding in spatiality [48].

Our study is not without limitations. First, identification of latent profiles is sensitive to sample size. It is possible that a fewer number of profiles would have emerged if we had limited the sample to only include the census tracts in which the CHEER sample lived. However, we included census tracts across the Denver-Metro and Northern Front Range of Colorado to obtain a more complete understanding of the neighborhood typologies in the study region. This study employed a cross-sectional design and thus we were unable to assess how changes in neighborhood environments impacted lung function, (e.g., due to gentrification). The small sample size of CHEER participants with acceptable spirometry curves (*n* = 182) limited our ability to detect associations between lung health and neighborhood profiles, but we believe the results are indicative of a true association between FEV_1_/FVC and residential neighborhoods. Although subjects with serious respiratory conditions (e.g., COPD) or contraindicators defined in the ATS guidelines [20] were not eligible to complete spirometry, it is possible that some CHEER subjects had undiagnosed respiratory diseases. Finally, by design, we sampled only low-income homes that qualified for federal programs or policies created to serve low-income populations, which means our study population may be generalizable to other low-income populations in the US, but may not be generalizable to higher income populations. Because the CHEER study sampled only low-income homes, we are able to estimate associations between respiratory health and neighborhood typologies within a population that, at the individual level, has similar socioeconomic statuses. Allowing for heterogeneity within a low-income population, the study design focuses on individuals at the highest risk of respiratory illness and should ideally net out the effect of individual SES on respiratory health, making the comparison across neighborhood typologies easier. Yet these associations may be conservative as the *sickest* CHEER participants (e.g., those with COPD) were unable to complete spirometry testing. Moreover, the CHEER study was designed to recruit low-income homes with a range of housing characteristics, which resulted in geographic variability in the types of neighborhoods in which participants lived.

## 5. Conclusions

Ours is the first study, to our knowledge, to examine the confluence of green space, traffic-related air pollution, violent and property crime, racial/ethnic composition, and socioeconomic status in different neighborhood typologies. Using latent profile analysis, we provided evidence that the health-enhancing or health-threatening aspects of social and environmental neighborhood contexts accumulate within typologies and cluster along lines of advantage/disadvantage. We have provided suggestive evidence of a double-jeopardy situation where low-income populations living in disadvantaged neighborhoods may have worse lung health. Using LPA with social factors and environmental hazards may help to identify neighborhoods that influence respiratory health, and better inform research on the complex and interdependent mechanisms by which neighborhoods influence health.

## Figures and Tables

**Table 1:** Characteristics of census-tract level latent profile analysis inputs by neighborhood typology.

LPA Input Variables	Mixed Race, High Poverty, Moderate-Poor Physical Environment	Hispanic and White, High Crime, Moderate Poverty, Poor Physical Environment	Hispanic and White, Moderate Poverty, Moderate Physical Environment	White, Wealthy, Good Physical Environment	Test of Difference	
**Physical Environment**					*F*	*p*
Green Space (mean NDVI)	0.24	0.22	0.27	0.28	50.94	<0.001
^1^ Annual Average NO_X_ (ppb)	36.00	60.48	17.20	5.70	204.97	<0.001
^1^ Annual Average PM_2.5_ (µg/m^3^)	2.84	7.12	1.33	0.43	218.5	<0.001
**Social Environment**					*F*	*p*
Violent Crime Rate (per 1000)	4	13	5	1	54.13	<0.001
Property Crime Rate (per 1000)	63	130	13	4	68.34	<0.001
Race/Ethnicity					*F*	*p*
Non-Hispanic White	46%	63%	72%	78%	92.69	<0.001
Non-Hispanic Black	12%	5%	1%	3%	94.53	<0.001
Hispanic	35%	25%	21%	12%	49.67	<0.001
**Socioeconomic Status**					*F*	*p*
Below Federal Poverty Level	19%	17%	15%	6%	61.57	<0.001
College Educated	33%	45%	40%	48%	17.14	<0.001
Homeowner	50%	48%	62%	77%	70.12	<0.001
Median Household Income	$51,285	$62,801	$61,866	$86,854	59.83	<0.001
Census Tracts in Study Area [*N* (%)]	139 (22%)	109 (17%)	178 (28%)	206 (33%)		

Test of Difference = ANOVA. *N* = 632 census tracts across the Denver-Metro and Front Range area of Colorado. Abbreviations: NDVI, normalized difference vegetation index; NO_x_, nitrogen oxides; PM_2.5_, fine particulate matter ^1^Annual average NO_x_ and PM_2.5_ values only include traffic-related sources.

**Table 2:** CHEER outcome and covariate characteristics and tests of difference by neighborhood typology.

Variable	Mixed Race, High Poverty, Moderate-Poor Physical Environment	Hispanic and White, High Crime, Moderate Poverty, Poor Physical Environment	Hispanic and White, Moderate Poverty, Moderate Physical Environment	White, Wealthy, Good Physical Environment	Test of Difference	
	Mean (SD)				*F*	*p*
FEV_1_ Z-Score	−0.63 (1.13)	−0.38 (1.08)	−0.31 (1.36)	−0.51 (1.17)	0.72	0.49
FVC Z-Score	−0.36 (1.06)	−0.31 (1.00)	0.05 (1.36)	−0.30 (1.05)	0.98	0.40
FEV_1_/FVC Z-Score	−0.50 (0.95)	−0.17 (0.95)	−0.60 (0.99)	−0.29 (0.86)	1.17	0.31
Age (years)	52 (20)	47 (17)	50 (22)	56 (20)	0.92	0.47
^1^ Annual Average Infiltration Rate	0.68 (0.34)	0.73 (0.35)	0.49 (0.22)	0.47 (0.16)	5.38	0.002
	%				X^2^	*p*
Sex (Female)	73%	64%	75%	70%	0.92	0.82
Non-Hispanic White	34%	23%	79%	52%	21.26	<0.001
Head of Household Reported Some College Education	48%	32%	79%	78%	17.67	<0.001
Never Smoker	66%	68%	69%	48%	1.75	0.63
Gas Stove in the Home	43%	50%	25%	13%	10.53	0.01
CHEER Participants [*N* (%)]	113 (62%)	22 (12%)	24 (13%)	23 (13%)		

*N* = 182 low-income individuals with 3 or more acceptable spirometric curves. Abbreviations: CHEER, Colorado Home Energy Efficiency and Respiratory Health study; FEV_1_, forced expiratory volume in 1 second; FVC, forced vital capacity. ^1^ Annual average household infiltration rate is a measure of home ventilation and equals the volume of indoor air replaced by outdoor air every hour given the usual method of home ventilation, averaged over the entire year, and expressed as air changes per hour.

**Table 3:** Generalized estimating equation models estimating FEV_1_, FVC, and FEV_1_/FVC z-scores as a function of neighborhood typologies.

Outcome by Neighborhood Typology	Unadjusted				^1^ Adjusted			
	Beta	SE	95% CI	*p*	Beta	SE	95% CI	*p*
FEV_1_ Z-Score								
Mixed Race, High Poverty, Moderate-Poor Physical Environment	−0.15	0.28	(−0.70, 0.39)	0.58	−0.13	0.29	(−0.70, 0.44)	0.65
Hispanic and White, High Crime, Moderate Poverty, Poor Physical Environment	0.07	0.34	(−0.60, 0.74)	0.84	0.12	0.38	(−0.63, 0.86)	0.76
Hispanic and White, Moderate Poverty, Moderate Physical Environment	0.11	0.42	(−0.71, 0.92)	0.79	0.26	0.37	(−0.47, 0.98)	0.49
White, Wealthy, Good Physical Environment	REF				REF			
FVC Z-Score								
Mixed Race, High Poverty, Moderate-Poor Physical Environment	−0.11	0.25	(−0.61, 0.38)	0.66	−0.07	0.34	(−0.63, 0.70)	0.91
Hispanic and White, High Crime, Moderate Poverty, Poor Physical Environment	−0.09	0.30	(−0.68, 0.51)	0.77	0.04	0.34	(−0.63, 0.70)	0.92
Hispanic and White, Moderate Poverty, Moderate Physical Environment	0.18	0.38	(−0.56, 0.93)	0.63	0.26	0.36	(−0.45, 0.96)	0.48
White, Wealthy, Good Physical Environment	REF				REF			
FEV/FVC Z-Score								
Mixed Race, High Poverty, Moderate-Poor Physical Environment	−0.21	0.19	(−0.58, 0.17)	0.28	−0.33	0.15	(−0.63, −0.02)	0.03
Hispanic and White, High Crime, Moderate Poverty, Poor Physical Environment	0.12	0.26	(−0.38, 0.63)	0.63	0.17	0.21	(−0.24, 0.59)	0.41
Hispanic and White, Moderate Poverty, Moderate Physical Environment	−0.31	0.29	(−0.87, 0.25)	0.28	−0.37	0.20	(−0.77, 0.03)	0.07
White, Wealthy, Good Physical Environment	REF				REF			

*N* = 182; 11 observations were excluded because of missing covariate values. Abbreviations: FEV_1_, forced expiratory volume in 1 second; FVC, forced vital capacity; SE, standard error; CI, confidence interval; REF, reference group. ^1^ Adjusted models include the following covariates: (1) binary race/ethnicity, (2) age categories, (3) sex, (4) binary indicator of head of household educational attainment, (5) binary indicator of smoking status (former vs never), (6) annual average household infiltration rate, (7) binary indicator of presence of gas stove in the home, and (8) binary indicator of lung function administered during flu season.
